# Castration-induced nigrostriatal deficits are linked to reduced TrkB and loss of mature spines in the dorsal striatum

**DOI:** 10.3389/fendo.2026.1828487

**Published:** 2026-06-03

**Authors:** Gerald J. Donahue, James A. Lundari, Patrick W. Kane, Samantha M. Matamorose-Patrick, Julia E. Klotz, Nicholas L. Carr, Stephen C. Kudriavetz, Joe C. Brague

**Affiliations:** 1 Neuroscience Program, University of Scranton, Scranton, PA, United States; 2 Department of Biology, University of Scranton, Scranton, PA, United States; 3 Department of Chemistry, University of Scranton, Scranton PA, United States

**Keywords:** castration, dendritic spines, Parkinson’s disease, testosterone, TrkB

## Abstract

Parkinson’s disease is a neurodegenerative disorder characterized by degeneration of nigrostriatal dopamine neurons and basal ganglia dysfunction, with sex differences in risk and progression that are not yet fully understood. Here, we investigated how peripubertal loss of gonadal hormones influences nigrostriatal integrity, striatal synaptic architecture, and motor behavior in male mice. Using a castration model, we show that early androgen deprivation recapitulates key neuropathological and behavioral features of Parkinsonian hypodopaminergia, including reduced tyrosine hydroxylase–positive neurons in the substantia nigra pars compacta, impaired motor performance on the vertical pole and rotarod, and disrupted forelimb kinematics. At the circuit level, androgen loss markedly reduced mushroom spine density on both direct- and indirect pathway striatal spiny projection neurons, with a concomitant increase in immature spine classes, consistent with impaired synaptic maturation. These structural and behavioral deficits were accompanied by reduced striatal Tyrosine receptor kinase-B (TrkB) protein, suggesting attenuation of brain derived neurotrophic factor (BDNF)/TrkB trophic signaling. Testosterone replacement preserved nigral dopaminergic neurons, normalized motor behavior, prevented mushroom spine loss across both striatal pathways, and selectively increased long/thin spines on direct-pathway neurons, suggesting enhanced synaptic plasticity. Together, these findings support a hormone-trophic model in which androgen signaling maintains nigrostriatal integrity and striatal synaptic maturation through androgen receptor-dependent BDNF/TrkB mechanisms. Although Parkinson’s disease predominantly manifests with aging, these results suggest that early disruption of androgen signaling can produce lasting circuit-level alterations that influence vulnerability to degeneration over time, providing mechanistic insight into sex differences in Parkinson’s Disease.

## Highlights

Parkinson’s disease occurs more frequently in men, yet the biological basis for this disparity is unclear. We show that loss of gonadal hormones during a critical developmental window weakens the nigrostriatal system by reducing dopamine neuron survival, impairing motor function, and destabilizing mature synapses in both major striatal pathways. These deficits coincide with decreased TrkB, a key receptor for BDNF signaling that supports neuronal health and synaptic maturation. Restoring gonadal hormones prevents dopamine neuron loss, preserves mushroom spines, and normalizes motor behavior. These findings identify androgen-dependent TrkB signaling as an essential regulator of striatal circuit stability and provide a mechanistic framework for understanding sex differences in Parkinson’s disease vulnerability and for exploring hormone or TrkB-based therapeutic strategies.

## Introduction

Parkinson’s disease (PD) is a progressive neurodegenerative disorder affecting over six million people worldwide (1). It is characterized by the loss of dopamine (DA) neurons in the substantia nigra pars compacta, leading to motor dysfunction. While age, heredity, and environmental toxins are well-established risk factors, *biological sex* (2) remains underexplored despite clear evidence of sex-based differences in PD prevalence, symptomology, and treatment outcomes (3, 4).

The dosal striatum plays a critical role in voluntary motor control. GABAergic spiny projection neuron (SPNs) are the major output neurons of the basal ganglia, which receive glutamatergic input from the cortex, thalamus, and dopaminergic input from the SNc. Direct pathway SPNs (dSPNs) express the dopamine 1 receptor and project to the substantia nigra pars reticulata (SNr) and the globus pallidus internus while indirect pathway SPNs (iSPNs) express the D2 receptor and project to the globus pallidus externus and subthalamic nucleus (5–8). The balance of iSPN and dSPN are critical for voluntary movement.

In PD, the loss of SNc dopamine input to the dorsal striatum impairs voluntary movement through activity changes in SPNs, shifting striatal output to favor iSPNs (9, 10). These changes manifest as altered excitatory connectivity to SPNs. Consistent with this loss, a reduction in mature spines and dendritic arbors of both SPN types has been observed in 6-OHDA rodent models and PD (11–14), the MPTP primate model (10, 13, 15), and postmortem brains of PD patients (9, 16). Therefore, strategies that enhance these connections, resulting in rebalanced striatal output, can be effective in alleviating PD motor deficits.

Khasnavis et al. (17) demonstrated that prepubertal loss of gonadal hormones induced PD pathologies, such as inflammation, activation of glial cells, degeneration of substantia nigra pars compacta (SNc), and motor impairments. The effects of this are driven largely through an increase in inducible nitric oxide synthase (iNOS) (18–20) and decrease in glial-derived neurotrophic factor (GDNF) (21, 22) in the substantia nigra, both of which are important to maintain SNc neuron integrity. While, this study is important in establishing a hormone-dependent model of PD, it highlights an important link between gonadal hormones, nigral degeneration, neurotrophic factors, and the loss of voluntary motor control.

Gonadal hormones regulate dendritic and synaptic architecture as well as neuronal function in steroid-sensitive brain regions, including the striatum, in part through modulation of neurotrophin signaling. Testosterone, in particular, maintains dendritic spine density across multiple neural circuits, such as the medial preoptic area (23), hippocampus (24), and basal ganglia (25, 26). Beyond morphology, testosterone dynamically modulates dopaminergic release and signaling within the striatum by acting on androgen receptors to influence presynaptic terminals and dopamine transporter expression (27). Androgen receptor signaling also regulates the neurotrophin receptor TrkB and its ligand, brain-derived neurotrophic factor (BDNF), thereby supporting neuronal structure and activity within the striatum (28, 29). Activation of TrkB by BDNF engages key intracellular signaling pathways—including PLCγ (30), RAS/MAPK (31), and PI3K/AKT (32), which promote neuronal survival, gene expression, growth, differentiation, and synaptic transmission (33).

The objective of this study is to determine how prepubertal gonadal hormone loss influences striatal circuitry and synaptic plasticity. DA depletion in the striatum reduces both mature and immature dendritic spine phenotypes on SPNs (9–16), while gonadal hormones enhance neurotrophic and ERK signaling (28, 29), and SPNs express both androgen and estrogen receptors (34, 35). Testosterone modulates nigrostriatal DA signaling via androgen receptor activation (36) and is also locally converted to estradiol by aromatase (27), enabling estrogenic regulation of synaptic structure and function. Based on this, we hypothesized that removal of gonadal hormones reduces both immature and mature spine phenotypes on dSPNs and iSPNs, accompanied by decreased TrkB and ERK signaling. Using a castration model, this study examines how gonadal hormone loss alters dendritic spine architecture, molecular markers of synaptic plasticity, and basal ganglia circuitry.

## Materials and methods

### Subject details and experimental design

Male C57BL/6 mice (The Jackson Laboratory, RRID: MGI:2159769) were housed in cages on a 12/12 light/dark cycle (7 A.M. lights on, 7 P.M. lights off). Animals had *ad libitum* access to food and water and were treated in compliance with the Institutional Care and Use Committee for the University of Scranton. Thirty mice were evenly and randomly placed into one of three conditions: 1) sham + empty silastic capsule (Sham), 2) Castration + empty silastic capsule (Cast), and 3) castration + testosterone silastic capsule (Cast+T). In each condition all mice received either a control or testosterone filled silastic implant, sham gonadectomy or gonadectomy, and stereotaxic injection of either saline or CTB-488 during the same surgery between P30 and P33. All animals underwent behavioral assays between P60 and P74. From each condition, three animals were euthanized for western blot, four for immunofluorescence, and three for DiL (1,1′-dioctadecyl-3,3,3′,3′-tetramethylindocarbocyanine) labeling. The only animals to receive CTB-488 into the SNr were those that underwent DiL labeling.

### Surgery

All surgical procedures were performed using aseptic technique. At 4–5 weeks of age, mice were weighed and anesthetized with a 3% induction/1-3% maintenance of isoflurane/100% oxygen gas mixture. Animals were checked for anesthetization by the loss of response to stimulation (toe pinch) and blink reflex. Animals were placed on a heating pad, shaved, and disinfected at the incision site with 70% ethanol, iodine, and again with 70% ethanol. Intra-operative isoflurane monitoring ensured respirations stayed within normal range during surgery.

#### Silastic implant

All mice received a single subcutaneous silastic implant placed near the right shoulder and oriented parallel to the spine. Implants consisted of 14 mm silicone tubing (0.04″ ID × 0.085″ OD). One end of the tubing was sealed with 2 mm silicone adhesive (Dow Corning) and weighed. The capsules were then loaded with either testosterone (Sigma T1500-5g) dissolved in 200-proof ethanol or an ethanol vehicle alone. Each testosterone containing capsule was loaded with 10ul of 200mg/ml and allowed to air dry for a total of 5 times or until the weight of the testosterone reached approximately 10mg. The other end of the capsule was then sealed, allowed to cure for 24 hours in the dark, and washed in 200-proof ethanol for 3 min immediately prior to implantation. This dosage yields approximately 8ng/ml concentration for up to 75 days (37). Sham and castrated mice received vehicle control implants (tubing that received a similar amount of ethanol without testosterone).

#### Gonadectomy

All animals were placed on a heating pad and an incision was made along the midline of the scrotum and the testes were exposed. For castrated animals, the vas-deferens and blood supply was ligated, then the testes were extracted. For all animals, the scrotum was then shut with sutures, and a thin layer of triple antibiotic ointment was added.

#### Stereotaxic injection

Anesthetized mice were placed in a stereotaxic apparatus on a heating pad, and sterile ophthalmic ointment was applied to the eyes. A 0.5–1.0 cm midline incision was made to expose the skull. Burr holes (0.70 mm) were drilled at bilateral coordinates (-3.28 AP, ± 1.25 ML, −4.60 DV). 1 µL of cholera toxin B-488 (CTB-488; ThermoFisher C22841) or sterile saline was infused at 0.2 µL/min using an internal 33-gauge cannula (C3151S-4; 6 mm projection) inserted into a 26-gauge guide cannula (C315GS-4; cut 0 mm below pedestal; Protech International), with 3-min incubation periods following cannula placement and injection. Following surgery, mice were sutured, administered ketoprofen (5 mg/kg; Zoetis), and allowed to recover for four weeks. Postoperative care included subcutaneous ketoprofen (5 mg/kg) for one day and saline injections (250 µL) for six days. Body weight was monitored, and mice losing >20% of presurgical weight were excluded. All surgeries (gonadectomy, silastic implant, and brain injection) took place at the same time in all animals.

### Behavior

Three behavioral assays were conducted: open field, vertical pole, and rotarod. All behavioral testing was conducted during the light phase between 9:00 A.M. and 5:00 P.M. Mice were acclimated to the testing room for 15 minutes prior to each behavioral session. On days involving multiple behavioral assays, a minimum inter-test interval of 15 min was maintained. The final day of each assay was designated as the testing day, and only data from testing days were included in analyses.

#### Open field

Mice were placed in a 15”x15”x15” plexiglass arena for 10 minutes for two consecutive days for acclimation followed by recording on day three using a GoPro HERO 11 (4k, 60fps). The enclosure was cleaned with 70% ethanol between trials. Open field videos were cropped to include only the testing arena using DaVinci Resolve 20 Software (Black Magic) and saved as a.MOV file. DeepLabCut 2.3.9 (DLC) (38) was used to track coordinate data of the nose, right forepaw, left forepaw, right hind paw, left hind paw, and the tail base. The network for locomotion kinematics was trained using 1391 labeled frames from nine videos (three per condition) for 1,200,000 iterations. X and Y coordinates of DLC tracking data were imported into Behavioral Segmentation of Open-field in DLC (B-SOiD) 2.0 (39). Following the steps described on the Github webpage (github.com/YttriLab/B-SOID). In short, B-SOiD was trained on a random subset of 20 files containing the pose estimation computed by DLC. The minimum cluster size range was set to 0.50 to 0.85 yielding 15 segmented behaviors. After identifying the forward locomotion segmented behavior (14) we ran the kinematics analysis feature that yielded cumulative probability plots for each fore and hind paw.

#### Vertical pole

The animal is placed head-up at the top of a rough surfaced 10mm diameter 50cm long metal pole covered in a plastic mesh. Mice were placed near the top of the pole facing upward. Time to reorient downward, time to descend to the bottom, and total trial time were recorded. Three trials were conducted per mouse with a 15 second rest period between trials. All animals underwent four days of acclimation followed by testing on day five.

#### Rotarod

Mice were tested on an accelerated rotarod paradigm. Animals underwent two days of acclimation, followed by testing on day three. During acclimation, each mouse completed three trials at a constant speed of 5 revolutions per minute (RPM) for 60 s; trials in which mice fell before 60 s were repeated, up to a maximum of four trials. On the test day, the rotarod accelerated from 5 to 60 RPM over 300 s. Latency to fall, rotational speed at fall, and total distance traveled were recorded.

### DiL labeling and immunohistochemistry

#### DiL labeling

To analyze spine morphology, three mice from each condition that were injected with bilateral CTB- 488 were euthanized with an overdose of ketamine/xylazine (200/20 mg/mL, respectively) and intracardially perfused with 25 mL PBS (7.95g NaCl, 0.20g KCl, 1.425g Na_2_HPO_4_, 0.27g KH_2_PO_4_), followed by 25 mL of 1.5% paraformaldehyde in PBS (para). The striatum was dissected and sectioned at 100µm on a vibratome (D.S.K., Microslicer DTK-1000) filled with cold PBS. Every other section was mounted onto microscope slides. Small DiL crystals (Invitrogen, D282) were placed on the dorsal striatum via glass micropipette. Sections were covered with PBS and incubated at 4 °C in the dark for 48 hours. Sections were then post-fixed in 4% para for 1 hour at room temperature before being washed in PBS, coverslipped, and mounted with DAPI Fluromount (Invitrogen, 00-4959-52). All remaining 100µm slices were fixed in 4% para before being stained for TH immunoreactivity. Slices were then mounted with DAPI Fluromount before being imaged on a confocal microscope (Evident Scientific, FV4000). Per condition, three to five dendrites from three separate neurons of the dorsal striatum were imaged at 60x oil immersion with Evident’s Cell Sens software, then individual spine types were manually counted in Fiji-ImageJ software.

#### Immunohistochemistry

The CTB-488 injected tissue containing the substantia nigra reticulata (SNr) were incubated in 30% sucrose overnight and 40 µm sections were cut on the cryostat (Leica, CM3050). SN slices were washed (3x5 minutes) in PBS and incubated in 1:1000 mouse α-tyrosine hydroxylase (Santa Cruz, sc-25269; RRID: AB_628422) with Triton X-100 in PBS for 24 hours at room temp. Sections were washed in PBS (3 × 5 min), then incubated for two hours at room temperature with 1:500 donkey α-mouse Alexa Fluor 647 (Jackson ImmunoResearch Labs, 711-605-152; RRID: AB_2492288) in PBS. Slices were then mounted with DAPI Fluromount before being imaged on a confocal microscope (Evident Scientific, FV4000).

Mice stereotaxically injected with saline in the SNr were euthanized with an overdose of ketamine/xylazine and intracardially perfused with 25 mL PBS, followed by 25 mL of 4% para. Following perfusion, brains were extracted and post-fixed in 4% para for 2 hours and 30% sucrose in PBS overnight at room temperature. Brains were then sectioned at 40µm on the Cryostat. Slices were washed (3x5 minutes) in PBS and incubated in 1:1000 mouse α-tyrosine hydroxylase (Santa Cruz, sc-25269; RRID: AB_628422) with Triton X-100 in PBS for 24 hours at room temp. Sections were washed in PBS (3 × 5 min), then incubated for 2 h at room temperature with 1:500 donkey α-mouse Alexa Fluor 488 (Jackson ImmunoResearch Labs, 711-545-152; RRID: AB_2313584) in PBS. Slices were then mounted with DAPI Fluromount before being imaged on the confocal (Evident Scientific, FV4000). Labeled neurons and terminals were imaged at 2x, 10x, and 20x with Evident’s Cell Sens software, then TH positive neurons were manually counted in Fiji-ImageJ software averaging nine slices of SN per animal.

### Western blot

#### Tissue prep

Mice were euthanized with ketamine/xylazine (200/20 mg/mL, respectively) and the Striatum and SN were dissected out and flash frozen. Tissues were sonicated in RIPA lysis buffer (ThermoFisher Scientific cat. #87787) and protease/phosphatase inhibitor (MP Biomedicals, 08W00017) until homogenous. The homogenate was centrifuged at 4°C for 45 minutes at 14000 RPM. The supernatant was removed and centrifuged for a subsequent 45 minutes under the same conditions. The final extracted supernatant was stored at -80°C until analysis.

#### BCA assay

Total protein was determined using a Thermo Scientific micro BCA assay kit (ThermoScientific, 23235). Standards used bovine serum albumin and RIPA lysis buffer and the kit was followed and applied to all samples. Standards and samples were run in triplicate on a Thermo Scientific Multiskan FC at 560 nm. Total concentrations of a sample’s replicates were averaged to determine total protein concentration.

#### Western blot

Samples from the SN and striatum were prepped with Laemmli buffer and diluted in water to create 20 μl solution containing 60 μg of protein each. Loading samples were incubated at 95 °C for 5 minutes. The Precision Plus Protein Dual Color Standards ladder (Bio-Rad, 1610374) was loaded into the first well. Samples from the Sham, Cast, and Cast+T conditions were loaded in the listed order and run in triplicate. Samples were separated in a 10% polyacrylamide gel run at 100 V for 10 minutes and then 200 V for 40 minutes at room temperature. The proteins were then transferred to a nitrocellulose membrane (Bio-Rad,1620112) on wet ice at 100 V for 80 min. Blots were then blocked in 5% milk/TBS-T solution for two hours at room temp. In general, blots were incubated in a single primary antibody rotating overnight, followed by a two-hour incubation in secondary conjugated HRP at 4°C, incubated in Clarity Western ECL Substrate (Bio-Rad, 1705060) for 5 minutes on each side at room temperature, and imaged with a Licor C-DiGit Blot scanner for chemiluminescence and also imaged on a BioRad GelDoc XR+ for colorimetric imaging. Blots were run through this series sequentially for all primary antibodies, including the loading control. The following are the series of blots. 1:2,000 mouse α-TH (Santa Cruz, sc-25269; RRID: AB_628422), 1:20,000 Donkey α-mouse HRP (Jackson ImmunoResearch, 715-035-150; RRID: AB_2340770), 1:1,000 mouse α-GAPDH (Santa Cruz, sc-32233; RRID: AB_627679), and 1:20,000 Donkey α-mouse HRP (Jackson ImmunoResearch, 715-035-150; RRID: AB_2340770). 1:1,000 mouse α-TrkB (Santa Cruz, sc-25269; RRID: AB_628422), 1:20,000 Donkey α-mouse HRP (Jackson ImmunoResearch, 715-035-150; RRID: AB_2340770), 1:1,000 mouse α-GAPDH (Santa Cruz, sc-32233; RRID : AB_627679), and 1:20,000 Donkey α-mouse HRP (Jackson ImmunoResearch, 715-035-150; RRID: AB_2340770). 1:1000 mouse α-ERK2 (Santa Cruz, sc-81458; RRID: AB_1122622), 1:20,000 Donkey α-mouse HRP (Jackson ImmunoResearch, 715-035-150; RRID: AB_2340770), 1:1,000 mouse α-GAPDH (Santa Cruz, sc-32233; RRID: AB_627679), and 1:20,000 Donkey α-mouse HRP (Jackson ImmunoResearch, 715-035-150; RRID: AB_2340770). All images were processed and analyzed using Image Lab software (BIORAD).

### Statistical analysis

All statistical analysis was conducted using RStudio and R4.5.2 software with statistical significance set to p=0.05. A Shapiro-Wilk test indicated all individual data sets had normal distributions, p<0.05. Code available upon request. For the following experiments, a one-way ANOVA with a Tukey’s Honestly Significant Difference (HSD) posthoc analysis was performed: TH positive cell counts, vertical pole, EPM, balance beam, and rotarod. For open field kinematics, a Klomogorov-Smirnov test was performed on the cumulative probability plots. For spines, a two-way analysis of variance (ANOVA) was conducted to determine the main effects of condition (hormonal state) and neuron type (D1 vs D2) on spine density. *Post hoc* comparisons were performed using HSD. Significance for all tests was denoted as *p<0.05, **p<0.01, or ***p<0.001.

## Results

### Immunoreactivity of TH positive neurons in the SN

To determine whether the loss of gonadal hormones reduced the number of TH positive neurons in the SN we counted the total number TH positive cells across nine slices of SN in five animals.

**Figure 1:**
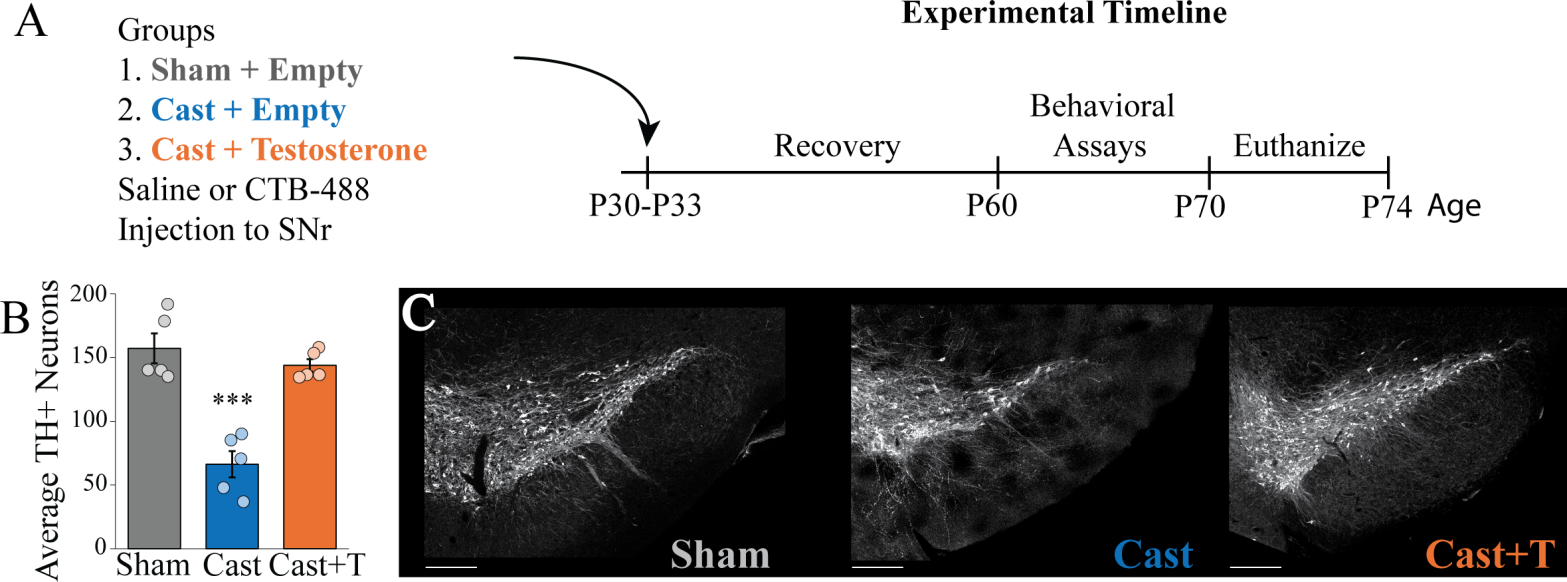
Experimental timeline and average TH^+^ SNc neuron count. **(A)** Experimental timeline. At P30-P33, Sham animals underwent a sham surgery, were implanted with empty silastic capsules, and received either saline or CTB-488 injections into the SNr. Castrated animals (Cast) were castrated, implanted with empty silastic capsules, and received either saline or CTB-488 injections into the SNr. Castrated + Testosterone (Cast+T) animals were castrated, given silastic capsules containing testosterone (T), and received either saline or CTB-488 injections into the SNr. Animals receiving CTB-488 were used for dendritic spine analysis and those injected with saline were utilized for the remaining experiments. **(B)** Average number of TH positive SNc neurons, B) Representative confocal microscope images of TH neurons in Sham, Cast, and Cast+T groups. Scale Bar 500μm. Data expressed as mean ± SEM ***P<0.001.

As reported previously (17), castrated animals have a reduced number of tyrosine hydroxylase (TH) positive neurons in the SNc (ANOVA:F(2,135)=48.6, p<0.001; HSD = p<0.001) when compared to Sham and Cast+T (**Figures 1A, B**) shows representative images of SN in Sham, Cast, and Cast+T.

### Motor behavior in a castration-based model of PD

**Figure 2:**
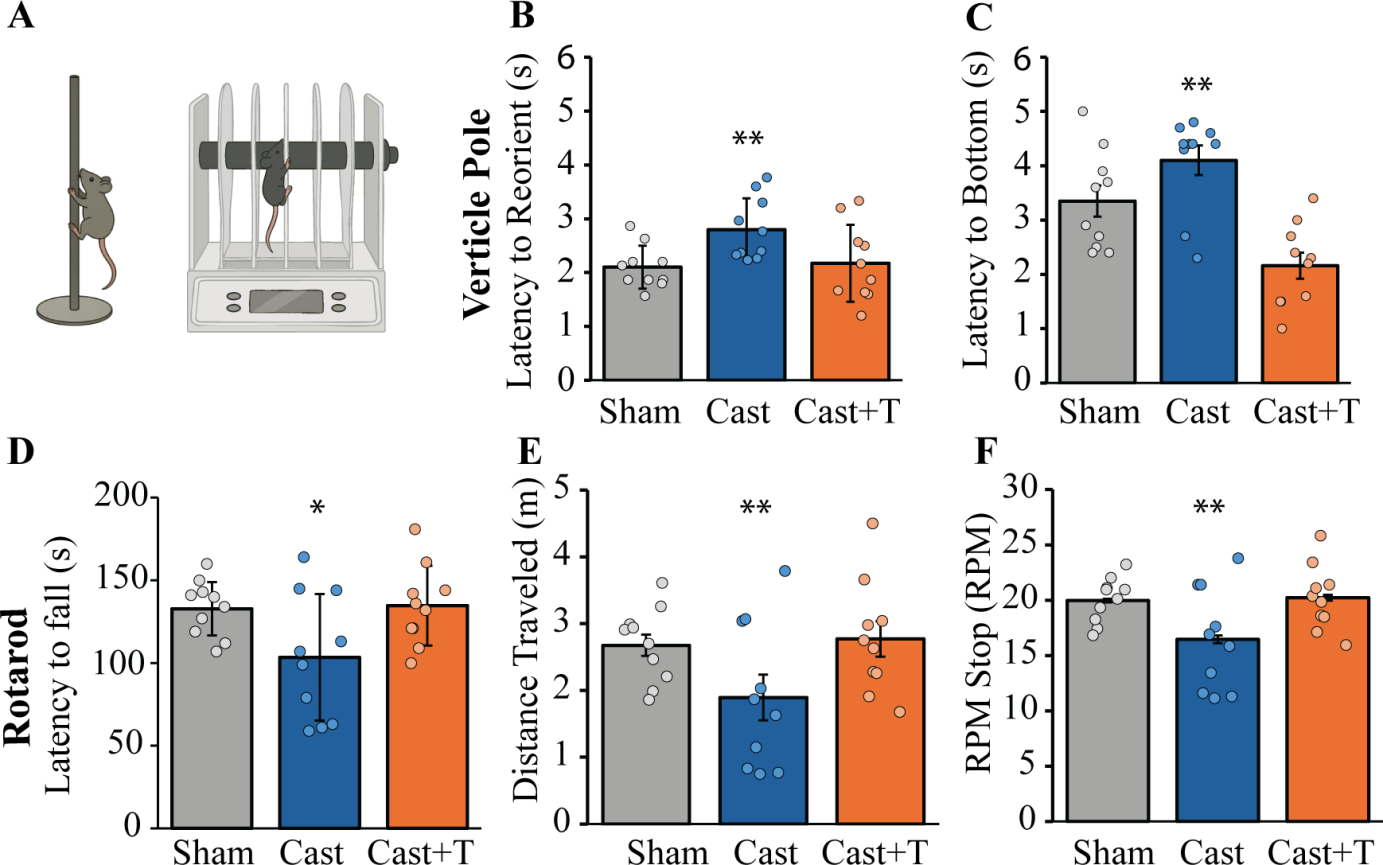
Vertical poll and rotarod motor behavior for Sham, Cast, and Cast+T. **(A)** cartoon images of the vertical poll and rotarod, respectively. **(B, C)** Latency to reorient downwards and latency to the bottom of the vertical poll. **(D–F)** Latency to fall, total distance traveled, and revolutions per minute (RPM) stop. Data expressed as mean ± SEM. *p<0.05, **p<0.01.

In additon, castrated animals have reduced motor activity and kinematics. **Figure 2A** shows a cartoon representation of a verticle poll and rotarod. Cast animals displayed a significant increase in the latency to reorient (F = 2,28)=15.9, p<0.01; HSD p<0.01) and latency to reach the bottom of the cage (F(2,28)=4.42, p<0.05; HSD p<0.01) (**Figures 2B, C**). On the rotarod, Cast animals again showed a decreased latency to fall (F(2,28)=4.16, p<0.05; HSD p<0.05), distanced traveled (F(2,28)=6.34, p<0.01; HSD p<0.01), and RPM stop time (F(2,28)=4.08, p<0.05; HSD p<0.01); (**Figures 2D–F**).

**Figure 3:**
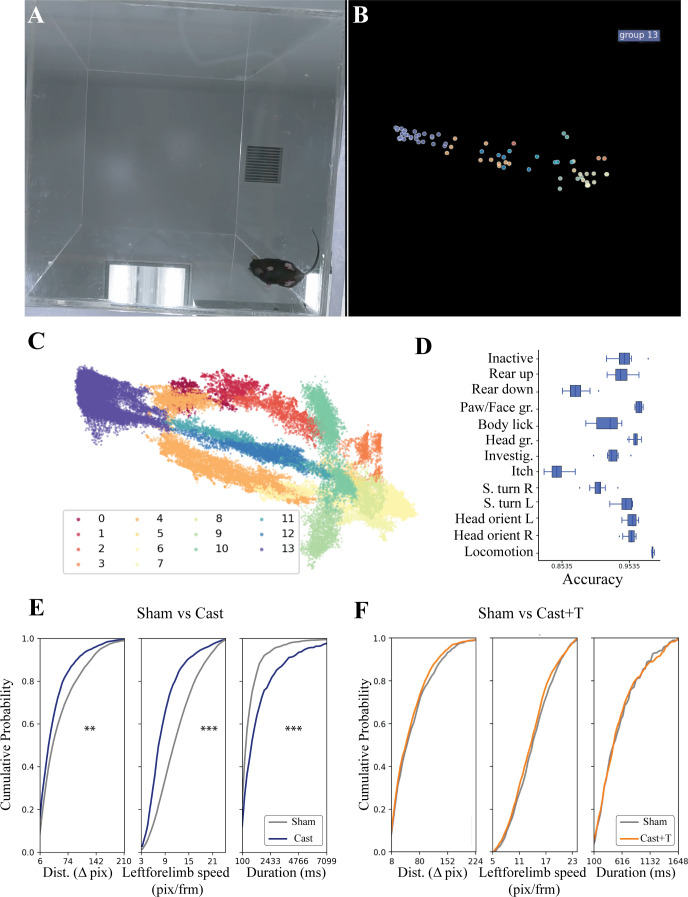
Open field analysis. **(A, B)** Example picture of a mouse in the open field arena with the corresponding B-SoiD behavior markers. **(C)** B-SOiD generated UMAP of behavior classifications. (S = stationary; R = right; L=left) **(D)** 10-fold group accuracy plot with behavior 13 being locomotion. **(E, F)** Cumulative distribution plot measuring left forelimb kinematics during locomotion when comparing Sham to Cast and Sham to Cast+T. **p<0.01 ***p<0.001.

Open field analysis indicated a reduction in distance, peak speed, and an increase in bout duration of forelimb kinematics in castrates (**Figure 3**). Following DLC tracking, B-SoiD generated a video snippet to interpret thirteen behavioral clusters (**Figures 3A, B**) organized onto a UMAP (**Figure 3C**). To ensure accuracy of tracking during locomotive bouts, a 10-fold cross-validation test yielded high accuracy (>95%) on shuffled data across the locomotion group. **Figure 3D**, E indicate that when comparing Sham to Cast, the Cast animals forelimb shows a reduced distance in Δpixels per frame (D(30)=.102, p<0.01), speed as pixels per frame (D(30)=.075, p<0.001), and an increase in bout duration (D(30)=.091, p<0.01). Conversely, there were no significant differences found when comparing Sham to Cast+T.

### dSPN and iSPN spine morphology analysis

**Figure 4:**
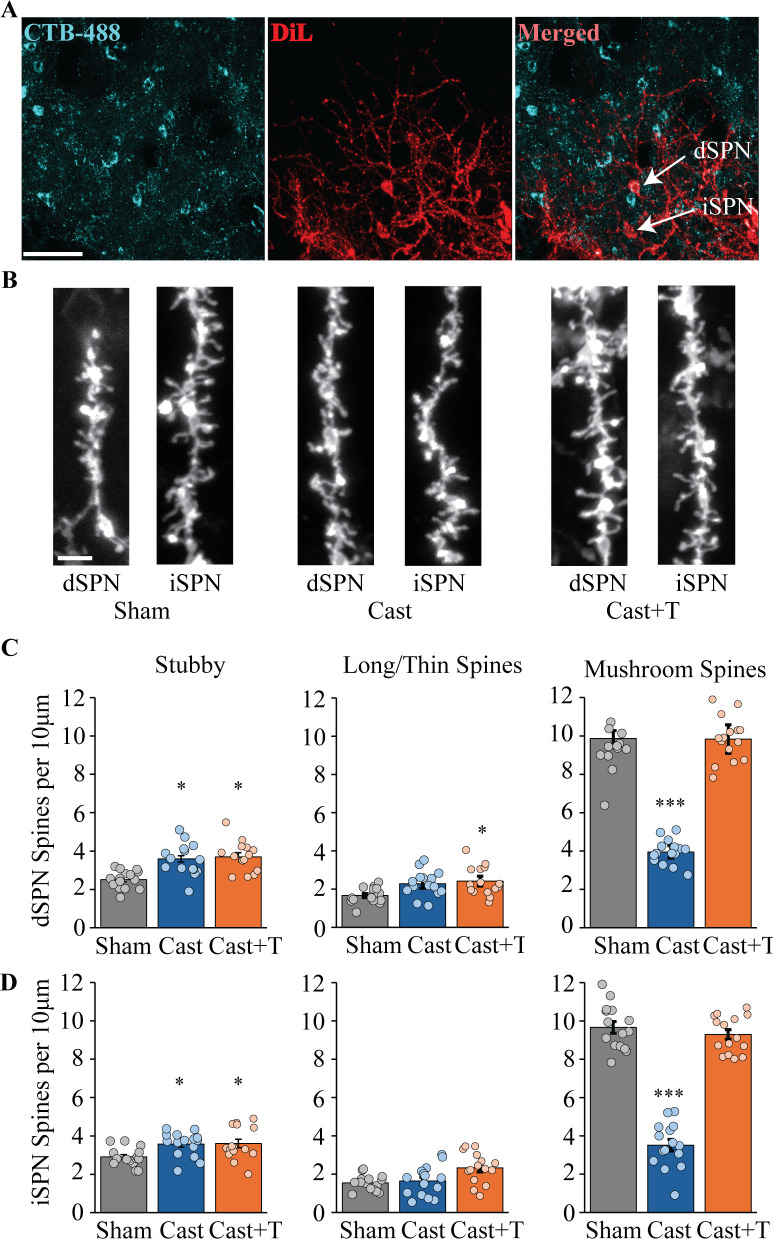
Striatal spine dynamics in a castration-based model of PD. **(A)** DiL crystal labeling of dSPN and iSPN neurons **(arrows)** with confocal microscope images of CTB-488, DiL, and Merged, respectively. Scale bar 50μm. **(B)** Representative spine images of dSPNs and iSPNs in the Sham, Cast, and Cast+T conditions. Scale Bar 3μm. **(C, D)** dSPN **(C)** and iSPN **(D)** spines per 10 μm for stubby, long/thin, and mushroom (left to right). Data expressed as mean ± SEM.*p<0.05 ***p<0.001.

To visualize dSPNs in striatal tissue we utilized the anatomy of the basal ganglia by first injecting the SNr with the retrograde tract tracer CTB-488 (**Figure 4A**); reliably differentiating dSPN from iSPN cell bodies in the striatum. High resolution dSPN/iSPN spine images were obtained from Sham, Cast, and Cast+T groups (**Figure 4B**). dSPN and iSPN spines were counted from each condition and classified into stubby, long/thin, and Mushroom (n=3 mice per condition, n=5 neurons per animal, and n=3 dendrites per neuron; **Figures 4C,D**).

A two-way ANOVA revealed a significant interaction between condition (Intact, Cast, and Cast+T) and spine type (stubby, long/thin, mushroom) F(4,252)= 170.863, p<0.001, ƞ_p_^2^ = 0.731. In dSPN neurons, there was an increase in stubby spines for Cast and Cast+T groups when compared to Sham (F(2,42)=11.08, p<0.001; HSD p<0.05). Long/thin spines showed an increase in Cast+T when compared to sham (F(2,42)=6.45, p<0.01; HSD p<0.05). The presence of testosterone was key in maintaining mushroom spines as Cast animals show a marked reduction in mushroom spines when compared to Sham and Cast+T (F(2,42)=118.15, p<0.001; HSD p<0.001). In iSPN neurons, there was an increase in stubby spines for Cast and Cast+T groups when compared to Sham (F(2,42)=7.29, p<0.01; HSD p<0.05). Long/thin spines showed no change in any condition. However, testosterone was key in maintaining mushroom spines as Cast animals show a marked reduction in mushroom spines when compared to Sham and Cast+T (F(2,42)=180.57, p<0.001; HSD p<0.001).

### Western blot analysis

**Figure 5:**
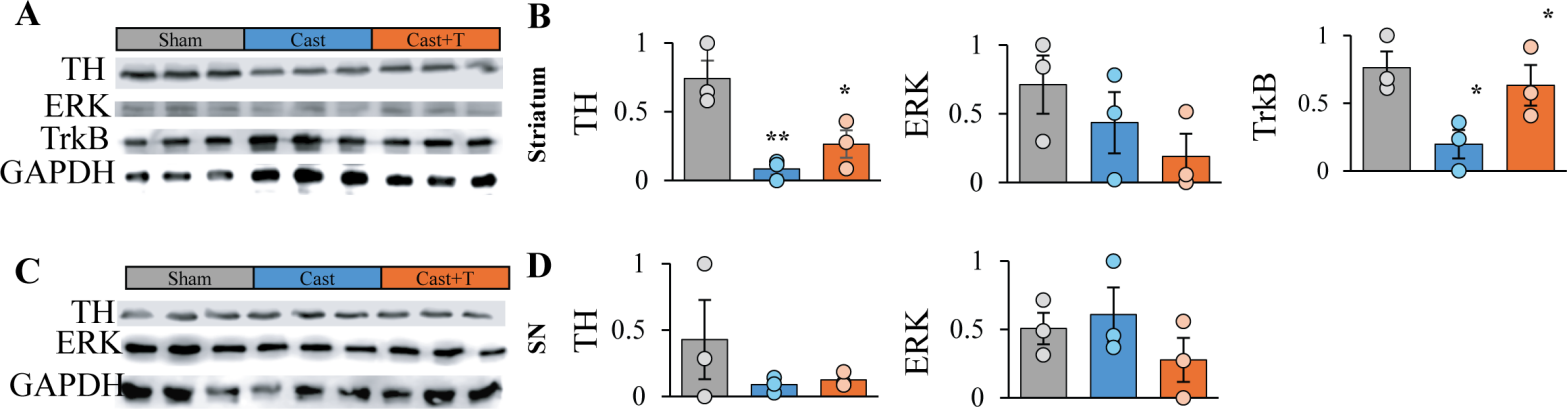
Western blot analysis of Sham, Cast, and Cast+T. **(A)** Striatal densitometry and **(B)** quantification of TH, ERK, and TrkB. **(C)** Substantia nigra (SN) densitometry and **(D)** quantification of TH, and ERK. Data expressed as mean ± SEM *p<0.05 **p<0.01.

Western blot analysis utilized an n=3 animals per condition with combined left/right striatum and SN. **Figures 5A, C** show the densitometry of TH, ERK, TrkB, and the loading control GAPDH. These data indicate that castration reduced protein levels of TH (F(2,6)=12.11, p<0.01, ƞ_p_^2^= 0.31.; HSD Sham vs Cast p<0.001; **Figure 5B**). In addition, when comparing Sham to Cast+T there was also a reduction (HSD p<0.05; **Figure 5B**). SN TH analysis indicated no significant differences between groups (**Figure 5D**). ERK2, a marker of synaptic plasticity, showed no significant differences in striatum or SN tissues (**Figures 5B, D**). Striatal TrkB however is reduced in castrated animals (F(2,6)=5.55, p<0.05, ƞ_p_^2^= 0.25.; HSD Sham vs Cast p<0.05 and Cast vs Cast+T p<0.05; **Figure 5D**).

## Discussion

Here we demonstrate that loss of gonadal hormones in young male mice recapitulates core neuropathological, cellular, and behavioral features of Parkinsonian hypodopaminergia, and that testosterone replacement is sufficient to partially rescue these deficits. Castration reduced the number of TH positive neurons in the SNc, impaired motor performance on the vertical pole and rotarod, and diminished forelimb locomotor kinematics. At the level of dorsal striatal circuitry, androgen loss markedly reduced mushroom spine density on both dSPN and iSPN pathways, while increasing immature spines, consistent with impaired synaptic maturation. These behavioral and cellular phenotypes were accompanied by a reduction in striatal TrkB protein, indicating attenuation of BDNF/TrkB trophic signaling likely secondary to dopaminergic neuron loss (40, 41). Testosterone replacement preserved SNc TH-positive neuron numbers, normalized motor behavior and forelimb kinematics, and prevented mushroom spine loss in both SPN populations. Additionally, testosterone increased the proportion of long/thin spines selectively on dSPNs, suggesting a shift toward a more plastic synaptic state. Together, these data support a mechanistic framework in which androgen signaling maintains dopaminergic neuron integrity and corticostriatal synaptic maturation, thereby preserving balanced basal ganglia output and normal motor control.

These findings extend earlier castration-based models of Parkinsonian pathology. Khasnavis and colleagues demonstrated that peripubertal gonadal hormone deprivation triggers neuroinflammation, elevates iNOS, reduces GDNF, and produces progressive SNc neurodegeneration accompanied by motor impairment (17–22). Our results recapitulate their major findings of SNc dopaminergic neuron loss following early androgen deprivation and directly link this pathology to deficits across multiple motor paradigms. Importantly, testosterone replacement prevented both SNc neuron loss and motor dysfunction, highlighting androgens as a critical protective factor during a developmental window when nigrostriatal circuits are still maturing. These data are consistent with epidemiological and clinical evidence that biological sex shapes PD prevalence, symptom profile, and treatment responsiveness (2–4). Furthermore, these data implicate an androgen-supported dopaminergic trophic tone and spine maintenance underlying these sex differences.

At the circuit level, testosterone is known to modulate striatal dopamine release, transporter expression, and BDNF/TrkB signaling through androgen receptor–dependent mechanisms (27–29). TrkB activation engages downstream PLCγ, RAS/MAPK, and PI3K/AKT signaling cascades that promote neuronal survival and synaptic growth (30–33). Consistent with this framework, androgen deprivation produced a robust decrease in striatal TrkB protein and a selective loss of mushroom spines (mature synapse) that confer synapse stability and sustained efficacy, in both dSPNs and iSPNs. Mushroom spine loss is a well-established hallmark of dopamine depletion in rodent (11–14) and primate models (10, 12, 15), as well as in postmortem PD tissue (9, 16), indicating that androgen loss recapitulates a canonical morphological feature of the Parkinsonian striatum.

Testosterone replacement prevented mushroom spine loss across both pathways while selectively increasing long/thin spines on dSPNs. Long/thin spines are thought to represent labile intermediates capable of either retraction or transition to mushroom spines depending on ongoing activity and trophic support (42). The enrichment of this spine class on dSPNs may reflect active remodeling toward mature synapses, consistent with restored motor performance. By contrast, the persistence of an elevated stubby spine fraction suggests incomplete normalization of the spine lifecycle, potentially reflecting timing, dosage, or pathway-specific thresholds for trophic signaling.

Although no group differences were detected in total ERK2 protein, this finding is consistent with the notion that ERK-mediated synaptic plasticity is controlled primarily through phosphorylation dynamics rather than changes in total protein abundance. Moreover, cell-type–specific or compartmentalized signaling changes may be obscured in bulk tissue analyses. Future studies incorporating phospho-TrkB and phospho-ERK measurements with cell-type resolution will be important for more directly linking androgen signaling to BDNF/TrkB pathway activation in striatal circuits.

Dopamine depletion is known to bias striatal output toward the indirect pathway, contributing to akinesia and bradykinesia. Loss of corticostriatal spines weakens excitatory drive, and although iSPNs often show preferential pruning in Parkinsonian models, dSPN changes are highly dependent on model, species, and disease stage (9–16). In the present study, androgen deprivation reduced mushroom spines in both SPN populations, consistent with a global weakening of corticostriatal efficacy that would be expected to impair movement initiation and vigor. These synaptic changes closely align with the observed deficits in balance, coordination, and forelimb kinematics revealed by B-SOiD analysis. The concurrent rescue of spine structure and motor behavior by testosterone suggests that androgen-dependent trophic signaling stabilizes corticostriatal synapses across both pathways while preferentially supporting direct-pathway throughput essential for normal motor output.

We observed a dissociation between SNc TH immunohistochemistry and bulk Western blot measurements. While castration reduced SNc TH-positive neuron counts that were restored by testosterone, total TH protein levels in SN samples did not differ significantly, whereas striatal TH protein, reflecting terminal integrity, remained reduced even after testosterone replacement. This discrepancy might be attributed to enhanced DA synthesis from the surviving SNc neurons (43), thereby showing increased TH in the sample. In addition, including SNr tissue in our samples may have diluted SNc-specific changes in the Sham and Cast+T groups. These effects highlight the need for longitudinal analyses with regionally enriched dissections and/or direct measures of dopamine content and release.

Together, these findings support a hormone–trophic model in which peripubertal androgens sustain nigrostriatal integrity and corticostriatal synaptic maturation through androgen receptor–BDNF/TrkB signaling. In this framework, peripubertal androgen loss reduces striatal DA and BDNF (44), and compromises spine maturation. Testosterone replacement restores trophic tone, stabilizes SPN mushroom spines, and preserves dopaminergic neurons, thereby rebalancing basal ganglia output and improving motor function. While Parkinson’s disease predominantly manifests with aging, early disruption of androgen signaling produce persistent alterations in nigrostriatal and corticostriatal circuit organization that influence vulnerability to degeneration over time. This model aligns with established sex differences in Parkinson’s disease and suggests that both developmental and age-related androgen decline may weaken dopaminergic and corticostriatal support systems critical for motor control.

In conclusion, peripubertal gonadal hormone loss is sufficient to induce cardinal features of Parkinsonian pathology, including SNc dopaminergic neuron loss, hypokinetic motor deficits, and pronounced disruption of striatal synaptic structure. These effects coincide with reduced TrkB signaling and are preventable by testosterone replacement. By linking endocrine status to trophic signaling, dendritic spine maturation, and basal ganglia circuit output, these results provide a mechanistic base for understanding sex differences in PD and suggest hormone- and TrkB-targeted strategies to mitigate motor dysfunction and, potentially, disease progression.

## Data Availability

The original contributions presented in the study are included in the article, further inquiries can be directed to the corresponding author.
